# Effects of the distant population density on spatial patterns of demographic dynamics

**DOI:** 10.1098/rsos.170391

**Published:** 2017-08-02

**Authors:** Kohei Tamura, Naoki Masuda

**Affiliations:** 1 Department of Engineering Mathematics, University of Bristol, Merchant Venturers Building, Woodland Road, Clifton, Bristol BS8 1UB, UK; 2 Frontier Research Institute for Interdisciplinary Sciences, Tohoku University, Aramaki aza Aoba 6-3, Aoba-ku, Sendai 980-8578, Japan

**Keywords:** demography, dynamics, gravity model, migration, population census

## Abstract

Spatio-temporal patterns of population changes within and across countries have various implications. Different geographical, demographic and econo-societal factors seem to contribute to migratory decisions made by individual inhabitants. Focusing on internal (i.e. domestic) migration, we ask whether individuals may take into account the information on the population density in distant locations to make migratory decisions. We analyse population census data in Japan recorded with a high spatial resolution (i.e. cells of size 500×500 *m*) for the entirety of the country, and simulate demographic dynamics induced by the gravity model and its variants. We show that, in the census data, the population growth rate in a cell is positively correlated with the population density in nearby cells up to a distance of 20 km as well as that of the focal cell. The ordinary gravity model does not capture this empirical observation. We then show that the empirical observation is better accounted for by extensions of the gravity model such that individuals are assumed to perceive the attractiveness, approximated by the population density, of the source or destination cell of migration as the spatial average over a circle of radius ≈1 km.

## 1. Introduction

Demography, particularly spatial patterns of population changes, has been a target of intensive research because of its economical and societal implications, such as difficulties in upkeep of infrastructure [1–3], policymaking related to city planning [1,2] and integration of municipalities [3]. A key factor shaping spatial patterns of demographic dynamics is migration. Migration decisions by inhabitants are affected by various factors including job opportunities, cost of living and climatic conditions [4–6]. These and other factors are often non-randomly distributed in space, creating spatial patterns of migration and population changes over time. A number of models have been proposed to describe and predict spatio-temporal patterns of human migration [7–13].

Among these models, a widely used model is the gravity model (GM) and its variants [8,10,14,15]. The GM assumes that the migration flow from one location to another is proportional to a power (or a different monotonic function) of the population at the source and destination locations and the distance between them. The model has attained reasonably accurate description of human migration in some cases [8,16,17], as well as other phenomena such as international trades [18,19] and the volume of phone calls between cities [20,21].

Studies of migration, such as those using the GM [8,17] and other migration models [11,22], are often based on subdivisions of the space that define the unit of analysis such as administrative units (e.g. country and city). However, the choice of the unit of analysis is often arbitrary. Humans whose migratory behaviour is to be modelled microscopically, statistically or otherwise, may pay less attention to such a unit than a model assumes when they make a decision to move home. This may be particularly so for internal (i.e. domestic) migrations rather than for international migrations because boundaries of administrative units may impact inhabitants less in the case of internal migrations than international migrations. This issue is related to the modifiable areal unit problem in geography, which stipulates that different units of analysis may provide different results [23]. For example, particular partitions of geographical areas can affect parameter estimates of gravity models [24]. To overcome such a problem, criteria for selecting appropriate units of analysis have been sought [24–28]. Another strategy to address the issue of the unit of analysis is to employ models with a maximally high spatial resolution. For example, a recently proposed continuous-space GM assumes that the unit of analysis is an infinitesimally small spatial segment [12]. This approach implicitly assumes that the unit of analysis, which a modelled individual perceives, is an infinitesimally small spatial segment. In fact, humans may regard a certain spatial region, which may be different from an administrative unit and have a certain finite but unknown size, as a spatial unit based on which they make a migration decision. If this is the case, individuals may make decisions by taking into account the environment in a neighbourhood of the current residence and/or the destination of the migration up to a certain distance. Here, we examine this possibility by combining data analysis and modelling, complementing past research on the choice of geographical units for understanding human migration [24–28].

In this paper, we analyse demographic data obtained from the population census of Japan carried out in 2005 and 2010, which are provided with a high spatial resolution [29]. We hypothesize that the growth rate of the population is influenced by the population density near the current location as well as that at the focal location, where each location is defined by a 500×500 *m* cell in the grid according to which the data are organized. We provide evidence in favour of this hypothesis through correlation-based data analysis. Then, we argue that the GM is insufficient to produce the empirically observed spatial patterns of the population growth. We provide extensions of the GM that better fit the empirical data, in which individuals are assumed to aggregate the population of nearby cells to calculate the attractiveness of the source or destination cell of migration.

## 2. Methods

### 2.1. Dataset

We analysed demographic dynamics using data from the population census in Japan [29], which consisted of measurements from *K*=1 944 711 cells of size 500×500 *m*. The census is conducted every 5 years. We used data from the censuses conducted in 2005 and 2010 because data with such a high spatial resolution over the entirety of Japan were only available for these years. We also ran the following analysis using the data from the census conducted in 2000 (appendix A), which were somewhat less accurate in counting the number of inhabitants in each cell than the data in 2005 and 2010 [30]. In the main text, we refer to the two time points 2005 and 2010 as *t*_1_ and *t*_2_, respectively. The number of inhabitants in cell *i* (1≤*i*≤*K*) at time *t* is denoted by *n*_*i*_(*t*). We used the latitude and longitude of the centroid of each cell to define its position. Basic statistics of the data at the three time points are presented in table 1.

**Table 1.** Statistics of the dataset.

year	2000	2005	2010
total population	126 925 843	127 767 994	128 057 352
average number of inhabitants in a cell	249.48	251.13	251.70
median number of inhabitants in a cell	33	41	38
number of populated cells	308 418	482 181	477 172

### 2.2. Spatial correlation

We defined the distance between cells *i* and *j*, denoted by *d*_*ij*_, as that between the centroids of the two cells in kilometres. We measured the spatial correlation in the number of inhabitants between a pair of cells at distance *d* by [31]

2.1 $$C(d)=\frac{1}{\sigma^{2}}\frac{\sum_{i^{\prime }=1}^{K^{\prime }}\sum_{j^{\prime }=1}^{K^{\prime }}(n_{i^{\prime }}-\bar{n})(n_{j^{\prime }}-\bar{n})I(d<d_{i^{\prime }j^{\prime }}\leq d+1)}{\sum_{i^{\prime }=1}^{K^{\prime }}\sum_{j^{\prime }=1}^{K^{\prime }}I(d<d_{i^{\prime }j^{\prime }}\leq d+1)},$$

which is essentially the Pearson correlation coefficient calculated from all pairs of cells at a distance ≈*d* apart. In equation (2.1), $\bar{n}=\sum_{i^{\prime }}^{K^{\prime }}n_{i^{\prime }}/K^{\prime }$ is the average number of inhabitants in an inhabited cell; $\sigma^{2}=\sum_{i}^{K^{\prime }}{\left(n_{i}-\bar{n}\right)}^{2}/K^{\prime }$ is the variance of the number of inhabitants in an inhabited cell; *I*(*d*<*d*_*i*^′^*j*^′^_≤*d*+1)=1 if *d*<*d*_*i*^′^*j*^′^_≤*d*+1 (*d*=0,1,2,…) and *I*(*d*<*d*_*i*^′^*j*^′^_≤*d*+1)=0 otherwise; *K*^′^ (=482 181 at time *t*_1_ and 477 172 at time *t*_2_) is the number of inhabited cells. In equation (2.1), the summations on the right-hand side are restricted to the inhabited cells *i*^′^ and *j*^′^. We suppressed the time in equation (2.1). It should be noted that *C*(*d*) can be larger than 1.

### 2.3. Correlation between the growth rate and the population density in nearby cells

In the analysis of the growth rate of cells described in this section, we only used focal cells *i* whose population size was between 10 and 100 at *t*_1_. We did so because the growth rate of less populated cells tended to fluctuate considerably and the growth rate of a more populated cell tended to be ≈0. We carried out the same set of analysis for cells whose population size was greater than 100 to confirm that the main results shown in the following sections remain qualitatively the same (appendix C). It should be noted that cell *i* may be partially water-surfaced.

To calculate the correlation between the rate of population growth in a cell and the population density in cells nearby, we first divided the entire map of Japan into square regions of approximately 50×50 km. The regions were tiled in a 64×45 grid to cover the whole of Japan. The minimum and maximum longitudes in the dataset were 122.94 and 153.98, respectively. Therefore, we divided the range of the longitude into 64 windows, i.e. [122.4, 123), [123, 123.5),…, [153.5, 154]. Similarly, the minimum and maximum latitudes were 45.5229 and 24.0604, respectively. We thus divided the range of the latitude into 45 windows, i.e. [24, 24,5), [24.5, 25),…, [45.5,46]. We classified each cell into one of the 64×45 regions on the basis of the coordinate of the centroid of the cell. Note that there were sea regions without any inhabitant. A region included 9600 cells at most.

The growth rate of cell *i* in the 5 years is given by

2.2 $$R_{i}=\frac{n_{i}(t_{2})-n_{i}(t_{1})}{n_{i}(t_{1})}.$$

We denoted by *D*_*i*_(*d*) the population density at time *t*_1_ averaged over the cells *j* whose distance from cell *i*, *d*_*ij*_, is approximately equal to *d*, i.e. *d*<*d*_*ij*_≤*d*+1. We calculated the Pearson correlation coefficient between the population growth rate (i.e. *R*_*i*_) and *D*_*i*_(*d*), restricted to the cells in region *k*, i.e.

2.3 $$\rho_{k}(d)=\frac{\sum_{i=1;\mathrm{cell}\;i\in \mathrm{region}\;k}^{K}(R_{i}-{\bar{R}}_{k})(D_{i}(d)-{\bar{D}}_{k}(d))}{\sqrt{\sum_{i=1;\mathrm{cell}\;i\in \mathrm{region}\;k}^{K}{\left(R_{i}-{\bar{R}}_{k}\right)}^{2}}\sqrt{\sum_{i=1;\mathrm{cell}\;i\in \mathrm{region}\;k}^{K}(D_{i}(d)-{\bar{D}}_{k}{\left(d)\right)}^{2}}},$$

where ${\bar{R}}_{k}$ and ${\bar{D}}_{k}(d)$ are the average of *R*_*i*_ and *D*_*i*_(*d*) over the cells in region *k*, respectively. A positive value of *ρ*_*k*_(*d*) is consistent with our hypothesis that the population growth rate is influenced by the population density in different cells. We remind that the summation in equation (2.3) is taken over the cells whose population is between 10 and 100. The correlation coefficient *ρ*_*k*_(*d*) ranges between −1 and 1. We did not exclude water-surface cells or partially water-surface cells *j* from the calculation of *D*_*i*_(*d*). Finally, we defined $\bar{\rho }(d)$ as the average of *ρ*_*k*_(*d*) over all regions excluding those with less than 20 populated cells. We decided to calculate *ρ*_*k*_(*d*) for individual regions, *k*, and averaged it over the regions rather than to calculate the single correlation coefficient between *R*_*i*_ and *D*_*i*_(*d*) for the entirety of Japan. In this way, we aimed to suppress fluctuations in individual *ρ*_*k*_(*d*). We show *ρ*_*k*_(*d*) for each region in appendix B. We also show *ρ*_*k*_(*d*) for region *k* such that all cells within region *k* and those within 30 km from any cell in region *k* are not in the sea in appendix B.

To examine the statistical significance of $\bar{\rho }(d)$, we carried out bootstrap tests by shuffling the number of inhabitants in the populated cells at *t*_2_ without shuffling that at *t*_1_ and calculating $\bar{\rho }(d)$. We generated 100 randomized samples and calculated the distribution of $\bar{\rho }(d)$ for each sample. We deemed the value of $\bar{\rho }(d)$ for the original data to be significant if it was not included in the 95% confidential interval (CI) calculated on the basis of the 100 randomized samples.

### 2.4. Gravity model

In the standard gravity model (GM), the migration flow from source cell *i* to destination cell *j* (≠*i*), *T*_*ij*_, is given by

2.4 $$T_{ij}=G\frac{n_{i}^{\alpha }n_{j}^{\beta }}{d_{ij}^{\gamma }},$$

where *G*, *α*, *β* and *γ* are parameters. Because *α*, *β* and *γ* are usually assumed to be positive, equation (2.4) implies that the migration flow is large when the source or the destination cell has many inhabitants or when the two cells are close to each other.

In addition to the GM, we investigated two extensions of the GM in which the migration flow depends on the numbers of inhabitants in a neighbourhood of cell *i* or *j*. The first extension, which we refer to as the GM with the spatially aggregated population density at the destination (d-aggregate GM), is given by

2.5 $$T_{ij}=G\frac{n_{i}^{\alpha }N_{j}{\left(d_{\mathrm{ag}}\right)}^{\beta }}{d_{ij}^{\gamma }},$$

where *N*_*j*_(*d*_ag_) is the number of inhabitants contained in the cells within distance *d*_ag_ km from cell *j*. We remind that the distance between two cells is defined as that between the centroids of the two cells. The rationale behind this extension and the next one is that humans may perceive the population density at the source or destination as a spatial average. A similar assumption was used in a model of city growth, where cells close to inhabitant cells were more likely to be inhabited [32].

The second extension of the GM aggregates the population density around the source cell. To derive this variant of the GM, we rewrite equation (2.4) as $T_{ij}=n_{i}\times n_{i}^{\alpha -1}n_{j}^{\beta }/d_{ij}^{\gamma }$ and interpret that each individual in cell *i* is subject to the rate of moving to cell *j*, i.e. $n_{i}^{\alpha -1}n_{j}^{\beta }/d_{ij}^{\gamma }$. The second extension, which we refer to as the GM with the aggregated population density at the source (s-aggregate GM), is defined by

2.6 $$T_{ij}=Gn_{i}\frac{N_{i}{\left(d_{\mathrm{ag}}\right)}^{\alpha -1}n_{j}^{\beta }}{d_{ij}^{\gamma }}.$$

Unless we state otherwise, we set *d*_ag_=0.65 in the d-aggregate and s-aggregate GMs, which is equivalent to the aggregation of a cell with the neighbouring four cells in the north, south, east and west. We will also examine larger *d*_ag_ values.

Using one of the three GMs, we projected the number of inhabitants in each cell at time *t*_2_ given the empirical data at time *t*_1_. The predicted number of inhabitants in cell *i* at time *t*_2_, denoted by ${\hat{n}}_{i}(t_{2})$, is given by

2.7 $${\hat{n}}_{i}(t_{2})=n_{i}(t_{1})+\sum_{j=1}^{K}T_{ji}-\sum_{j=1}^{K}T_{ij}.$$

We refer to $\sum_{j=1}^{K}T_{ji}$, $\sum_{i=1}^{K}T_{ij}$ and $\sum_{j=1}^{K}T_{ji}-\sum_{j=1}^{N}T_{ij}$ as the inflow, outflow and net flow of the population at cell *i*, respectively.

The projection of the growth rate, denoted by ${\hat{R}}_{i}$, is defined by ${\hat{R}}_{i}=[{\hat{n}}_{i}(t_{2})-n_{i}(t_{1})]/n_{i}(t_{1})=(\sum_{j=1}^{K}T_{ji}-\sum_{j=1}^{K}T_{ij})/n_{i}(t_{1})$, based on which we calculated $\bar{\rho }(d)$ for the model. We set *G*=1 because the value of $\bar{\rho }(d)$ does not depend on *G*.

We measured the discrepancy between the empirical and projected data in terms of $\bar{\rho }(d)$ by

2.8 $$\frac{\sum_{d=0}^{99}|{\bar{\rho }}_{\mathrm{empirical}}(d)-{\bar{\rho }}_{\mathrm{model}}(d)|}{\sum_{d=0}^{99}|{\bar{\rho }}_{\mathrm{empirical}}(d)|},$$

where ${\bar{\rho }}_{\mathrm{empirical}}(d)$ and ${\bar{\rho }}_{\mathrm{model}}(d)$ are the values of $\bar{\rho }(d)$ obtained for the empirical data and a model, respectively. If the relationship between *ρ*(*d*) and *d* is similar between the empirical data and the model, the discrepancy given by equation (2.8) takes a small value.

## 3. Results

### 3.1. Spatial distribution of inhabitants

**Figure 1.**
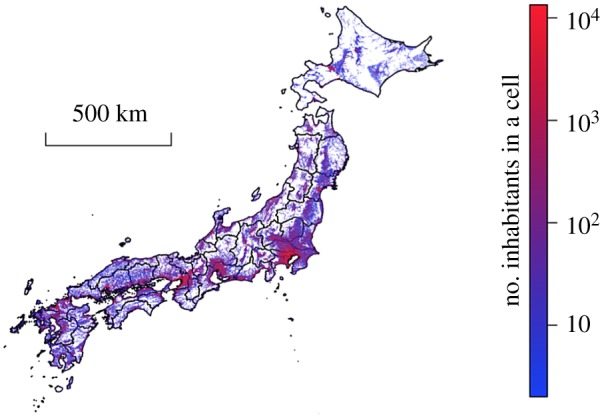
The distribution of inhabitants at time *t*_2_ (i.e. year 2010). The colour code represents the numbers of inhabitants in a cell. Vacant cells are shown in white.

**Figure 2.**
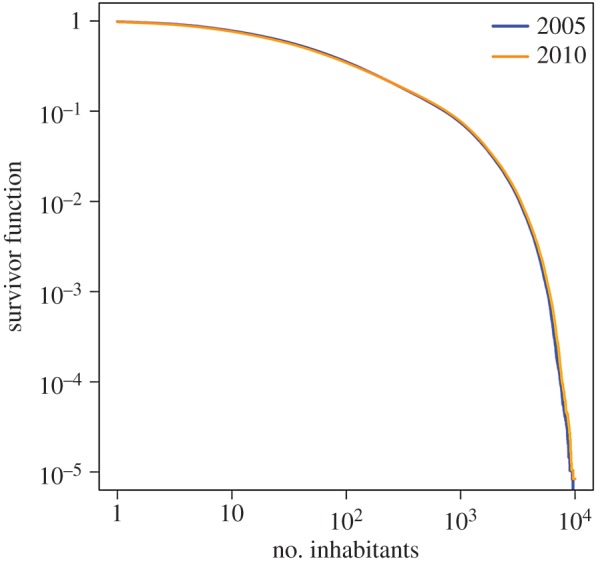
The survivor function of the number of inhabitants in a cell. The two lines almost overlap with each other.

The spatial distribution of the number of inhabitants at time *t*_2_ is shown in figure 1. The figure suggests centralization of the number of inhabitants in urban areas. We calculated the Gini index, defined by $1/2K^{{\;}^{\prime }2}\times \sum_{i^{\prime }=1}^{K^{\prime }}\sum_{j^{\prime }=1}^{K^{\prime }}|n_{i^{\prime }}-n_{j^{\prime }}|/\bar{n}$, to quantify heterogeneity in the population density across cells; it is often used for measuring wealth inequality. The Gini index at *t*_1_ and *t*_2_ was equal to 0.797 and 0.804, respectively, suggesting a high degree of heterogeneity. The survival function of the number of inhabitants in a cell at *t*_1_ and *t*_2_ is shown in figure 2. The figure suggests that a majority of cells contains a relatively small number of inhabitants, whereas a small fraction of cells has many inhabitants.

**Figure 3.**
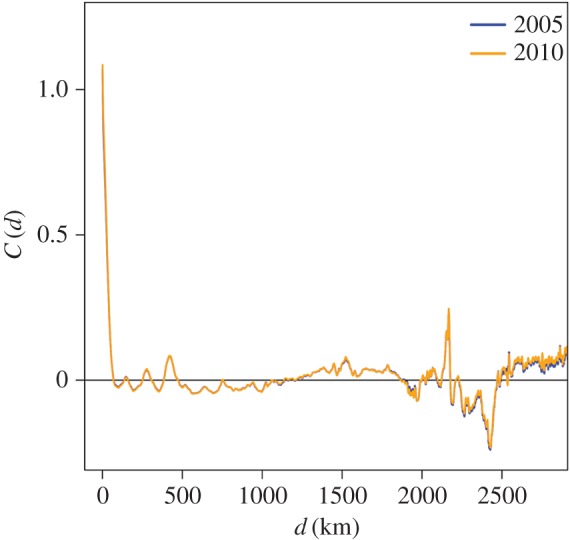
The spatial correlation in the number of inhabitants in the cell. The correlation measure *C*(*d*) is defined by equation (2.1), and *d* is the distance between the two cells. The two lines almost overlap with each other.

Figure 1 suggests the presence of spatial correlation in the population density, as observed in other countries [31]. Therefore, we measured the spatial correlation coefficient in the population size between a pair of cells, *C*(*d*), where *d* was the distance between a pair of cells. Figure 3 indicates that *C*(*d*) is substantially positive up to *d*≈70 km, confirming the presence of spatial correlation. This correlation length was shorter than that observed in previous studies of data recorded in the USA [31] (≈1000 km) and spatial correlation in the population growth rate in Spain [33] (≈500 km) and the USA [34] (over 5000 km).

### 3.2. Effects of the population density in nearby cells on migration

**Figure 4.**
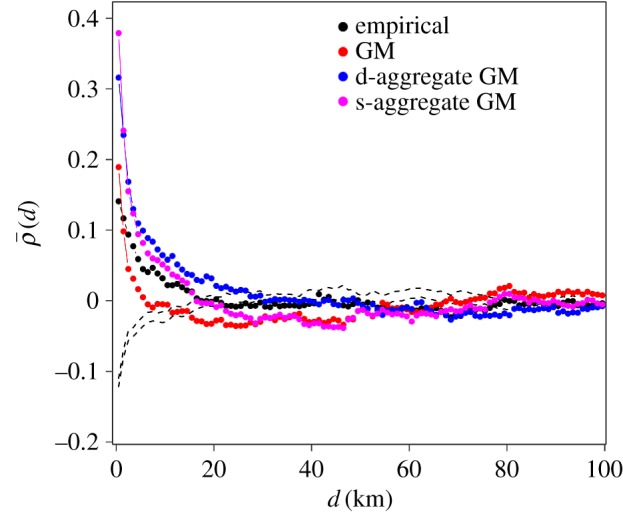
Dependence of the population growth rate in a cell on the population density at distance *d*, $\bar{\rho }(d)$. We set *α*=0.4, *β*=0.8 and *γ*=1 for the GM; *α*=0.8, *β*=0.4, *γ*=1 and *d*_ag_=0.65 km for the d-aggregate GM; *α*=0.4, *β*=0.4, *γ*=1 and *d*_ag_=0.65 km for the s-aggregate GM. The ranges indicated by the dashed lines represent 95% confidence intervals (CIs) generated by spatially random distributions of the number of inhabitants on the inhabited cells.

We measured $\bar{\rho }(d)$, which quantifies the effect of the population in cells at distance *d* on the population growth in a focal cell. Figure 4 shows $\bar{\rho }(d)$ as a function of *d*. The values of $\bar{\rho }(d)$ were the largest at *d*=0. In other words, the effects of the population density within 1 km is the most positively correlated with the growth rate of a cell. This result reflects the observation that highly populated cells tend to grow and vice versa [35–37] (but see [38]). As *d* increased, $\bar{\rho }(d)$ decreased and reached ≈0 for *d*≥20 km. This result suggests that cells surrounded by cells with a large (small) population density within ≈20 km are more likely to gain (lose) inhabitants.

The observed correlation between the population growth rate of a cell and the population of nearby cells may be explained by the combination of spatial correlation in the population density (figure 3) and positive correlation between the population growth rate and the population density in the same cell. To exclude this possibility, we measured $\bar{\rho }(d)$ as the partial correlation coefficient, modifying equation (2.3), controlling for the population size of a focal cell. The results were qualitatively the same as those based on the Pearson correlation coefficient (appendix D).

### 3.3. Gravity models

Various mechanisms may generate the dependence of the population growth rate in a cell on different cells (up to ≈20 km apart), including heterogeneous birth and death rates that are spatially correlated. Here, we focused on the effects of migration as a possible mechanism to generate such a dependency. We simulated migration dynamics using the gravity model [8,10,15] and its variants and compared the projection obtained from the models with the empirical data. We did not consider the radiation models [11,12] including intervening opportunity models [7] because our aim here was to qualitatively understand some key factors that may explain the effects of distant cells observed in figure 4 rather than to reveal physical laws governing migration.

In figure 4, we compare $\bar{\rho }(d)$ between the empirical data and those generated by the GM, d-aggregate GM and s-aggregate GM. Because precise optimization is computationally too costly, we set *γ*=1 and set *α*, *β*∈{0.4,0.8,1.2,1.6} to search for the optimal pair of *α* and *β*. For this parameter set, all models yielded positive values of $\bar{\rho }(0)$, consistent with the empirical data. For the GM, $\bar{\rho }(d)$ decreased towards zero as *d* increased for *d*<6 km, i.e. the value of $\bar{\rho }(d)$ decayed faster than the empirical values. At *d*>6 km, $\bar{\rho }(d)$ generated by the GM was around zero but tended to be smaller than the empirical values. The two extended GMs yielded a decay of $\bar{\rho }(d)$, which hit zero at *d*≈20 km, qualitatively the same as the behaviour of the empirical data. The two extended GMs generated larger $\bar{\rho }(d)$ values than the empirical values for *d*≤20 km.

**Figure 5.**
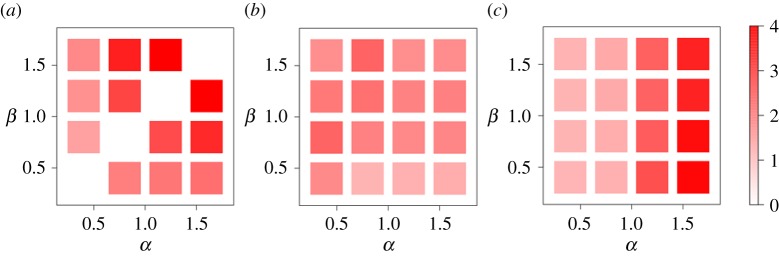
The discrepancy of the GM, d-aggregate GM and s-aggregate GM from the empirical data in terms of the discrepancy measure given by equation (2.8). A dark hue represents a large discrepancy value. (*a*) GM. (*b*) d-aggregate GM. (*c*) s-aggregate GM. The diagonals in (*a*) are blank because the inflow and outflow are the same when *α*=*β* in the GM, resulting in a zero population growth rate in all cells. We set *γ*=1 and *d*_ag_=0.65 km.

To investigate the robustness of the results against variation in the parameters of the models, we varied the parameter values as *α*∈{0.4,0.8,1.2,1.6} and *β*∈{0.4,0.8,1.2,1.6} and measured the discrepancy between the model and empirical data in terms of the discrepancy measure defined by equation (2.8). The results for the three models are shown in figure 5. The data obtained from the GM were inaccurate except when *α* or *β* was small. In addition, the minimum discrepancy for the GM (=1.469) was larger than that for the d-aggregate GM and s-aggregate GM (=1.163 and 1.161, respectively). The d-aggregate GM showed a relatively good agreement with the empirical data in a wide parameter region. The performance of the s-aggregate GM was comparable with that of the d-aggregate GM only when *α*=0.4 or 0.8. Our analysis suggests that aggregating nearby cells around either the source or destination of migration seems to improve the explanatory power of the GM. The performance of the d-aggregate GM was better than that of the s-aggregate GM in terms of the robustness against variation in the parameter values.

### 3.4. Effects of the granularity of spatial aggregation

**Figure 6.**
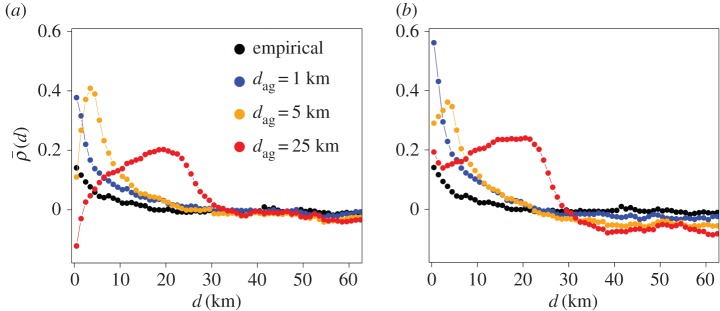
Dependence of the population growth rate in a cell on the population density at distance *d*, $\bar{\rho }(d)$, calculated from the empirical and numerical datafor different values of *d*_ag_. (*a*) d-aggregate GM. We set *α*=0.8, *β*=0.4 and *γ*=1.0. (*b*) s-aggregate GM. We set *α*=0.4, *β*=0.4 and *γ*=1.0.

We set *d*_ag_, the width for spatial smoothing of the population density at the source or destination cell in the extended GM models, to 0.65 km in the previous sections. To investigate the robustness of the results with respect to the *d*_ag_ value, we used *d*_ag_=1, 5 and 25 km combined with the d-aggregate and s-aggregate GMs. The discrepancy between each model and the empirical data is shown in figure 6.

When *d*_ag_=1 km, for both models, the results were similar to those for *d*_ag_=0.65 km (figure 4). When *d*_ag_=5 and 25 km, the behaviour of $\bar{\rho }(d)$ was qualitatively different, with $\bar{\rho }(d)$ first increasing and then decreasing as *d* increased, or even more complicated behaviour (i.e. s-aggregate GM with *d*_ag_=25 km shown in figure 6*b*).

**Figure 7.**
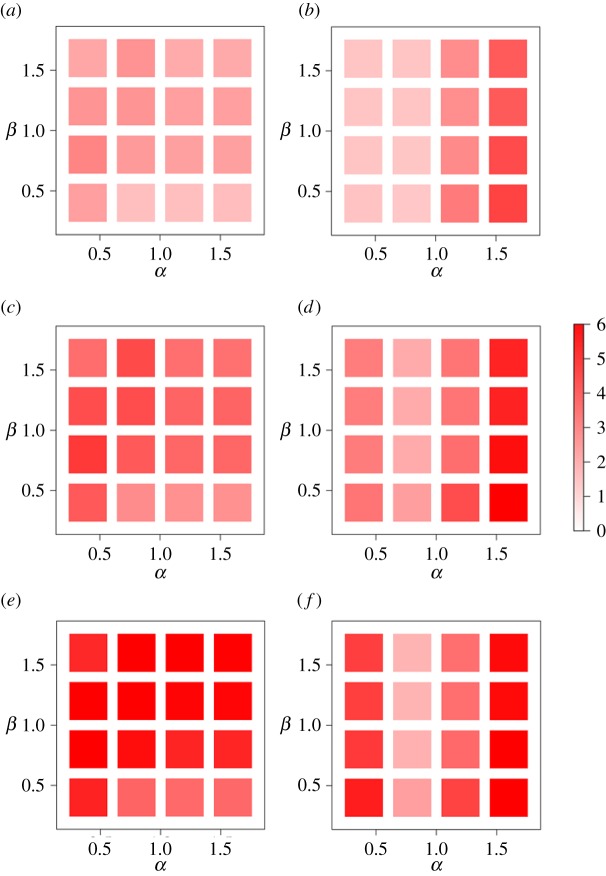
The discrepancy of d-aggregate GM and s-aggregate GM from the empirical data. (*a*) d-aggregate GM, *d*_ag_=1 km. (*b*) s-aggregate GM, *d*_ag_=1 km. (*c*) d-aggregate GM, *d*_ag_=5 km. (*d*) s-aggregate GM, *d*_ag_=5 km. (*e*) d-aggregate GM, *d*_ag_=25 km. (*f*) s-aggregate GM, *d*_ag_=25 km.

Figure 7 confirms that the results shown in figure 6 remain qualitatively the same in a wide range of *α* and *β*. In other words, the results for *d*_ag_=1 (figure 7*a*,*b*) are similar to those for *d*_ag_=0.65 (figure 5*b*,*c*), whereas those for *d*_ag_=5 (figure 7*c*,*d*) and *d*_ag_=25 (figure 7*e*,*f*) are not. We conclude that aggregating the population density at the source or destination of migration with *d*_ag_=5 km or larger does not even qualitatively explain the empirical data.

### 3.5. One-dimensional toy model

**Figure 8.**
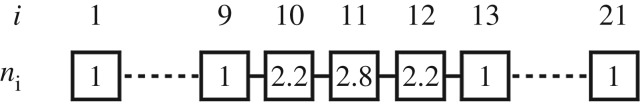
The schematic of the GM models on a chain. A square represents a cell, and *n*_*i*_ is the initial number of inhabitants in cell *i*. We set *x*=2.8 and *x*′=2.2 for illustration.

To gain further insights into the spatial inter-dependency of the population growth rate in terms of in- and out-migratory flows of populations, we analysed a toy model on the one-dimensional lattice (i.e. chain) with 21 cells (figure 8). Differently from the simulations presented in the previous sections, the current toy model assumes a flat initial population density except in the three central cells. Combined with the simplifying assumption of the one-dimensional landscape, we aimed at revealing a minimal set of conditions under which the empirically observed patterns were produced. We focused on the central cell and its two neighbouring cells, one on each side on the chain. We set the initial number of inhabitants in the central cell to *x*, those of the two neighbouring cells to *x*′ and those of the other cells to one as normalization. The distance between two adjacent cells was set to unity without a loss of generality. Then, we investigated the net flow (i.e. population growth rate), inflow and outflow of populations as a function of *x* and *x*′ using the three GMs. We set *d*_ag_=1, with which we aggregated three cells to calculate the population density at the source or destination of the immigration in the two extensions of the GM.

**Figure 9.**
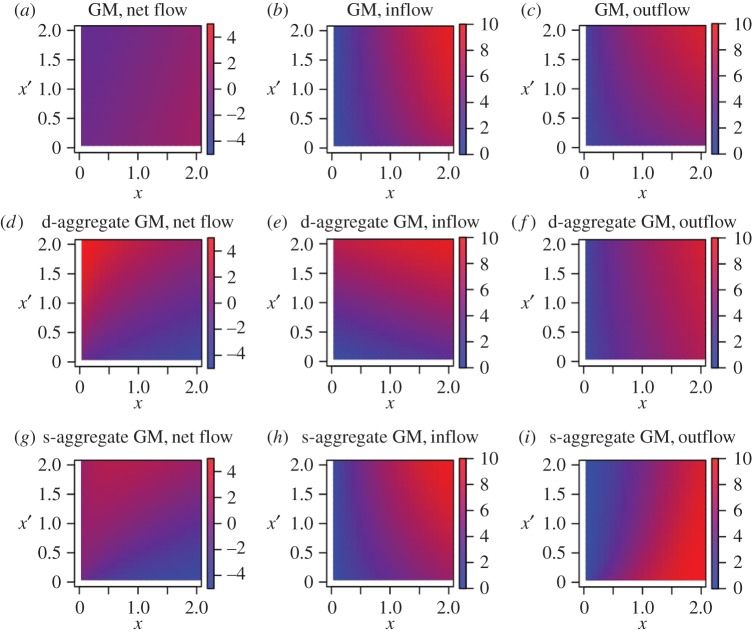
The net flow, inflow and outflow for the GM, d-aggregate GM and s-aggregate GM in the one-dimensional model with 21 cells. The initial condition is a symmetric distribution of the density of inhabitants that is uniform except in the central three cells. The initial population density is equal to *x* in the central cell, *x*′ in the neighbouring two cells and 1 in the other cells. We set *G* for the GM, d-aggregate GM and s-aggregate GM to 1, ${\left(\frac{1}{3}\right)}^{\beta }$ and ${\left(\frac{1}{3}\right)}^{\alpha -1}$, respectively, and *α*=0.4, *β*=0.6 and *γ*=1.0. (*a*) Net flow for the GM. (*b*) Inflow for the GM. (*c*) Outflow for the GM. (*d*) Net flow for the d-aggregate GM. (*e*) Inflow for the d-aggregate GM. (*f*) Outflow for the d-aggregate GM. (*g*) Net flow for the s-aggregate GM. (*h*) Inflow for the s-aggregate GM. (*i*) Outflow for the s-aggregate GM.

The net flow, inflow and outflow in the three models are shown in figure 9. In the GM, the net flow at the central cell heavily depended on *x* but negatively and only slightly depended on the population size in the neighbouring cells *x*′ (figure 9*a*). This result was inconsistent with the empirically observed pattern (figure 4). This inconsistency was due to an increase in the outflow at the central cell as *x*′ increased (figure 9*c*), whereas the inflow at the central cell was not sensitive to *x*′ (figure 9*b*).

The patterns of migration flows for the d-aggregate and s-aggregate GMs were qualitatively different from those for the GM (figure 9*d*–*i*). In both models, the population growth rate increased as *x*′ increased (figure 9*d*,*g*), which is consistent with the empirically observed patterns. In the d-aggregate GM, this change was mainly owing to changes in the inflow, which increased as *x*′ increased (figure 9*e*). The outflow for the d-aggregate GM was similar to that for the GM (figure 9*f*). In other words, a cell surrounded by those with higher population density attracted a larger migration flow in the d-aggregate GM. By contrast, in the s-aggregate GM, changes in the population flow were mainly attributed to changes in the outflow. The inflow for the d-aggregate GM was similar to that for the GM (figure 9*h*) and the outflow decreased as *x*′ increased for the d-aggregate GM (figure 9*i*). In other words, a cell surrounded by those with higher population density was less likely to lose inhabitants in the s-aggregate GM.

### 3.6. Gravity model with the aggregation around both the source and destination cells

**Figure 18.**
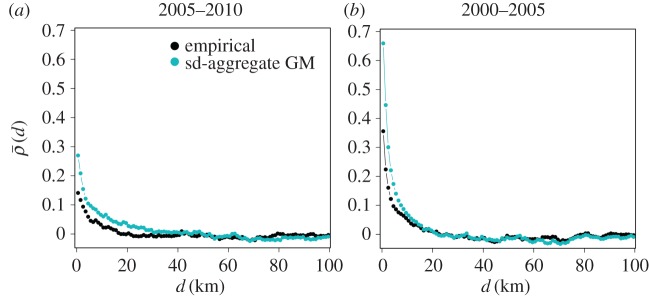
Dependence of the population growth rate in a cell on the population density at distance *d*, $\bar{\rho }(d)$, calculated from the empirical data and the numerical datagenerated from the sd-aggregate GM. (*a*) (*t*_1_,*t*_2_)=(2005,2010). We set *α*=0.4, *β*=1.6, *γ*=1 and *d*_ag_=0.65 km. (*b*) (*t*_1_,*t*_2_)=(2000,2005). We set *α*=0.4, *β*=0.4, *γ*=1 and *d*_ag_=0.65 km.

**Figure 19.**
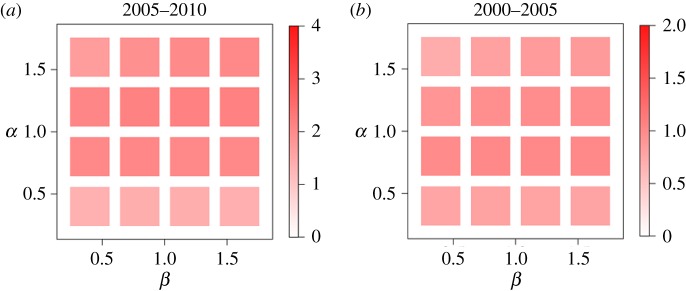
The discrepancy of the sd-aggregate GM from the empirical data. (*a*) (*t*_1_,*t*_2_)=(2005,2010). (*b*) (*t*_1_,*t*_2_)=(2000,2005). We set *γ*=1 and *d*_ag_= 0.65 km.

**Figure 20.**
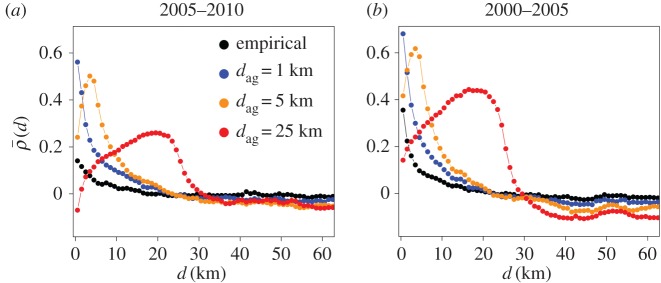
Dependence of the population growth rate in a cell on the population density at distance *d*, $\bar{\rho }(d)$, calculated for the sd-aggregate GM for different valuesof *d*_ag_. (*a*) (*t*_1_,*t*_2_)=(2005,2010). We set *α*=0.4, *β*=1.6 and *γ*=1.0. (*b*) (*t*_1_,*t*_2_)=(2000,2005). We set *α*=0.4, *β*=0.4 and *γ*=1.0.

**Figure 21.**
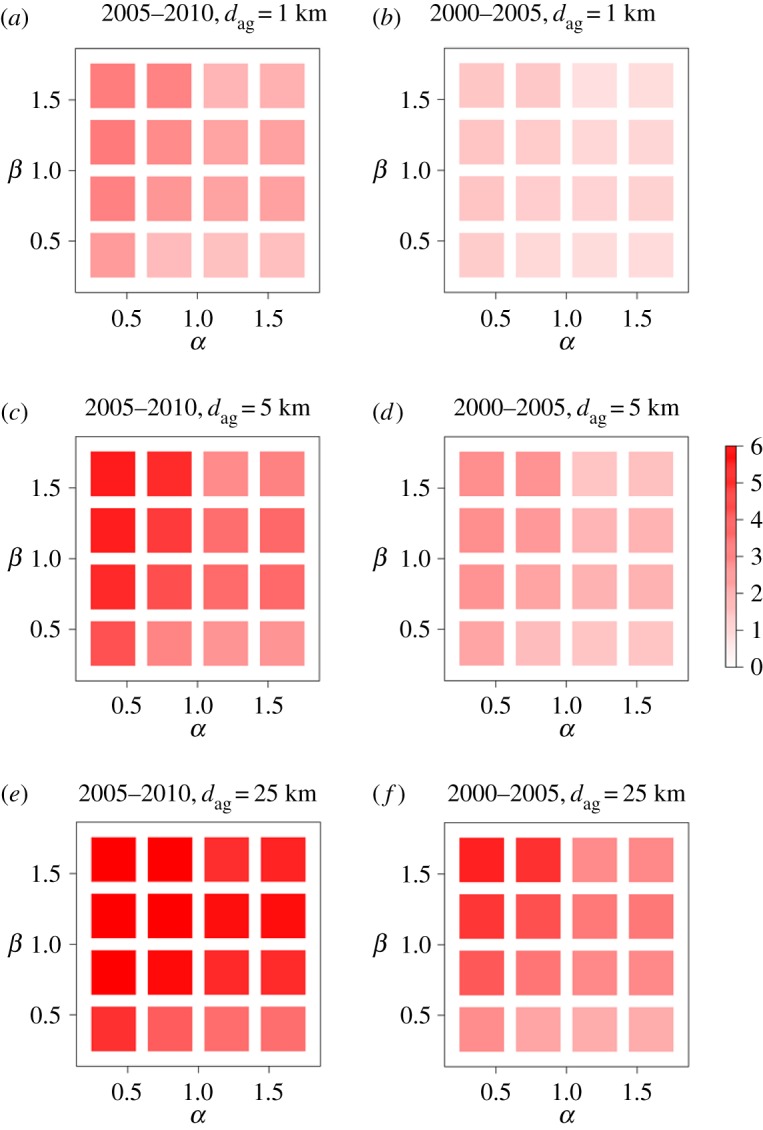
The discrepancy of the sd-aggregate GM from the empirical data. (*a*) (*t*_1_,*t*_2_)=(2005,2010), *d*_ag_=1 km. (*b*) (*t*_1_,*t*_2_)=(2000,2005), *d*_ag_=1 km. (*c*) (*t*_1_,*t*_2_)=(2005,2010), *d*_ag_=5 km. (*d*) (*t*_1_,*t*_2_)= (2000,2005), *d*_ag_=5 km. (*e*) (*t*_1_,*t*_2_)=(2005,2010), *d*_ag_=25 km. (*f*) (*t*_1_,*t*_2_)=(2000,2005), *d*_ag_=25 km.

**Figure 22.**
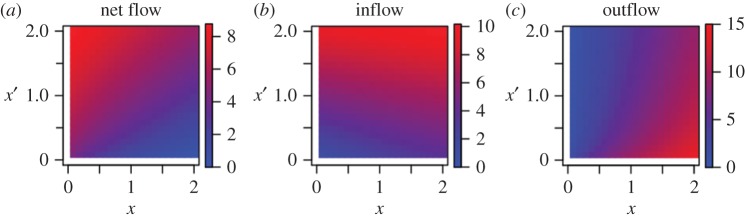
(*a*) The net flow, (*b*) inflow and (*c*) outflow for the sd-aggregate GM in the one-dimensional model. We set $G={\left(\frac{1}{3}\right)}^{\alpha +\beta -1}$, *α*=0.4, *β*=0.6 and *γ*=1.0.

Lastly, we investigated an extension of the GM with the aggregation of cells around both the source and destination cells, called the sd-aggregate GM (appendix E). The behaviour of $\bar{\rho }(d)$ was qualitatively the same as that obtained from the d-aggregate GM, s-aggregate GM and empirical data (figure 18). In addition, the sd-aggregate GM was accurate in a wide parameter region (figure 19). We also confirmed that the discrepancy measure for the sd-aggregate GM increased as *d*_ag_ increased (figures 20 and 21), similar to the results for the d-aggregate and s-aggregate GMs (figures 6 and 7). The behaviour of this model on the one-dimensional toy model was also consistent with the empirical data (figure 22) because the inflow and outflow of the model were similar to those for the d-aggregate GM and s-aggregate GM, respectively.

## 4. Discussion

We investigated spatial patterns of demographic dynamics through the analysis of the population census data in Japan in 2005 and 2010. We found that the population growth rate in a cell was positively correlated with the population density in cells nearby, in addition to that in the focal cell. We used the gravity model and its variants to investigate possible effects of migration on the empirically observed spatial patterns of the population growth rate. Under the framework of the GM, we found that aggregating some neighbouring cells around either the source or destination of migration events considerably improved the fit of the GM model to the empirical data. The results were better when the cells around the destination cell were aggregated, in particular regarding the robustness of the results against variation in the parameter values, than when the cells around the source cell were aggregated. All the results were qualitatively the same when we set *t*_1_=2000 and *t*_2_=2005, although the census data in 2000 were less accurate than those in 2005 and 2010 (appendix A).

Aggregation of cells near the destination cell models behaviour of individuals that perceive the population of the destination cell as a sum (or average) of the population over the cells neighbouring the destination cell. Because the size of the cell is imposed by the empirical data, aggregation of cells around the destination cell is equivalent to decreasing the spatial resolution of the GM by coarse graining. Traditionally, administrative boundaries have been used as operational units of the GM [39]. A cluster identified by the city clustering algorithm may also be used as the unit [38,40]. In the continuous-space GM, the unit is assumed to be an infinitely small spatial segment [12]. However, there is no *a priori* reason to assume that any one of these units is an appropriate choice. Our results suggest that spatial averaging with a circle of radius *d*_ag_≈1 km may be a reasonable choice as compared to a larger *d*_ag_ or the original cell size (i.e. 500×500 *m*^2^). Real inhabitants may perceive the population density at the destination as a spatial average on this scale. Although we reached this conclusion using the GMs, this guideline may be also useful when other migration models are used.

The present study has limitations. First, due to a high computational cost, we only examined a limited number of combinations of parameter values in the GMs. A more exhaustive search of the parameter space or the use of different migration models, as well as analysing different datasets, warrants future work.

Second, due to the lack of empirical data, we could not analyse more microscopic processes contributing to population changes. For example, because of the absence of spatially explicit data on the number of births and deaths, we did not include births and deaths into our models. However, the observed inflow and outflow were at least twice as large as the numbers of births and deaths in all the 47 prefectures in Japan (table 2). Therefore, migration rather than births and deaths seems to be a main driver of spatially untangled population changes in Japan during the observation period. The lack of data also prohibited us from looking into the effect of the age of inhabitants. In fact, individuals at a certain life stage are more likely to migrate in general [4,5]. Data on migration flows between cells, births, deaths and the age distribution, which are not included in the present dataset, are expected to enable further investigations of the spatial patterns of population changes examined in the present study.

**Table 2.** The number of births, deaths, incoming inhabitants and outgoing inhabitants in the 47 prefectures in Japan between 2005 and 2009. The relative contribution of migration to demographic dynamics, denoted by RC in the table, is defined by (inflow + outflow)/(the number of births + the number of deaths + inflow + outflow). The average of RC over the 47 prefectures is 0.801. Data were obtained from refs. [41–45].

prefecture	births	deaths	inflow	outflow	RC
Hokkaido	206 018	258 620	1 400 785	1 486 704	0.861
Aomori	50 846	75 402	210 797	253 252	0.786
Iwate	51 415	74 612	208 183	237 908	0.780
Miyagi	98 143	101 836	569 239	590 600	0.853
Akita	37 200	68 304	132 922	161 071	0.736
Yamagata	45 724	67 185	162 073	182 237	0.753
Fukushima	84 844	106 394	299 166	337 621	0.769
Ibaraki	123 337	134 098	539 995	543 333	0.808
Tochigi	85 950	91 337	338 813	343 879	0.794
Gunma	84 469	93 531	315 194	323 801	0.782
Saitama	302 796	251 786	1 673 676	1 612 124	0.856
Chiba	259 317	230 495	1 573 082	1 479 729	0.862
Tokyo	518 801	481 388	4 228 697	3 855 587	0.890
Kanagawa	393 305	307 305	2 434 444	2 297 378	0.871
Nigata	92 614	123 745	274 628	301 798	0.727
Toyama	43 760	56 352	134 116	142 212	0.734
Ishikawa	50 547	52 753	181 465	190 238	0.783
Fukui	35 888	39 657	100 717	111 694	0.738
Yamanashi	34 806	42 633	155 103	166 314	0.806
Nagano	91 097	109 115	369 322	389 224	0.791
Gifu	88 156	95 085	323 422	344 227	0.785
Shizuoka	163 151	165 452	702 346	710 330	0.811
Aichi	347 947	269 444	1 679 203	1 602 590	0.842
Mie	78 283	87 013	305 184	310 744	0.788
Shiga	66 776	54 067	271 852	260 071	0.815
Kyoto	108 329	113 508	605 810	623 003	0.847
Osaka	383 123	354 039	2 038 886	2 050 292	0.847
Hyogo	241 991	239 518	1 092 350	1 099 138	0.820
Nara	55 617	59 986	248 748	271 018	0.818
Wakayama	38 888	57 061	134 100	153 523	0.750
Tottori	24 973	32 708	89 781	101 313	0.768
Shimane	28 992	43 489	110 090	122 946	0.763
Okayama	84 959	93 940	306 532	316 719	0.777
Hiroshima	127 862	131 562	601 952	613 915	0.824
Yamaguchi	57 910	83 418	248 565	266 357	0.785
Tokushima	30 023	43 472	122 127	133 197	0.776
Kagawa	42 961	52 403	173 267	182 174	0.788
Ehime	57 940	76 292	213 144	231 838	0.768
Kochi	28 744	45 893	122 876	139 076	0.778
Fukuoka	228 884	220 437	1 308 177	1 312 289	0.854
Saga	38 216	43 612	151 729	163 207	0.794
Nagasaki	60 771	76 205	264 659	306 307	0.807
Kumamoto	80 457	91 365	333 680	351 123	0.799
Oita	50 366	61 697	208 513	216 427	0.791
Miyazaki	50 795	57 628	226 740	244 558	0.813
Kagoshima	75 607	96 695	369 723	399 374	0.817
Okinawa	82 886	47 009	368 805	373 402	0.851

Third, our conclusions are based on the longitudinal data at only two time points in a single country. The strength of the current results should be understood as such.

Fourth, we did not take into account the effect of water-surface cells, which cannot be inhabited. The population density at distance *d* from a focal cell *i*, i.e. *D*_*i*_(*d*), is therefore underestimated when cell *i* is located near water (e.g. sea, lake, large river). Additional information about the geographical property of cells such as the water area within the cell and the land use may improve the present analysis.

## Figures and Tables

**Figure 10.**
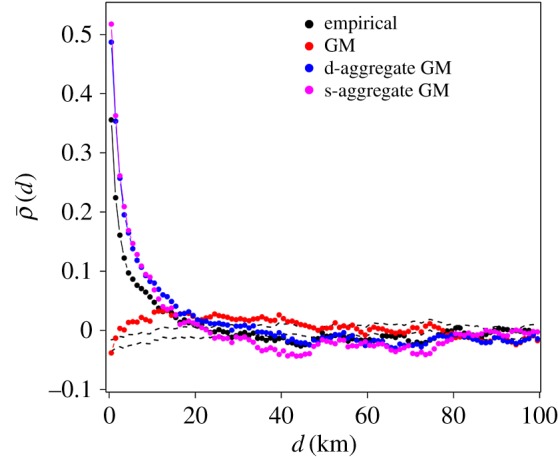
Dependence of the population growth rate in a cell on the population density at distance *d*, $\bar{\rho }(d)$, calculated from the empirical and numerical data between 2000 and 2005. We set *α*=0.8, *β*=0.4 and *γ*=1 for the GM; *α*=0.8, *β*=0.4, *γ*=1 and *d*_ag_=0.65 km for the d-aggregate GM; and *α*=0.4, *β*=1.2, *γ*=1 and *d*_ag_=0.65 km for the s-aggregate GM. The ranges indicated by the dashed lines represent 95% CIs.

**Figure 11.**
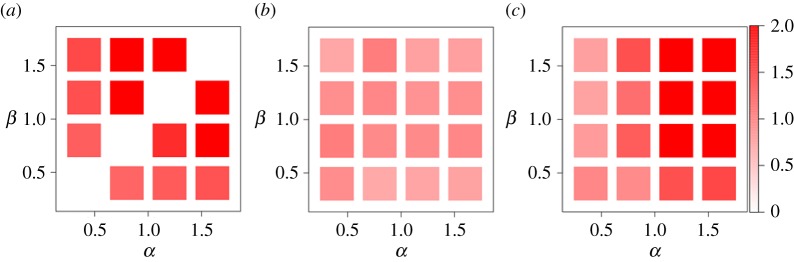
The discrepancy of the GM, d-aggregate GM and s-aggregate GM from the empirical data, calculated for the population change between 2000 and 2005. (*a*) GM. (*b*) d-aggregate GM. (*c*) s-aggregate GM. The diagonal in (*a*) is blank because the inflow and outflow are equal when *α*=*β* in the GM, resulting in no population change. We set *γ*=1 and *d*_ag_=0.65 km.

**Figure 12.**
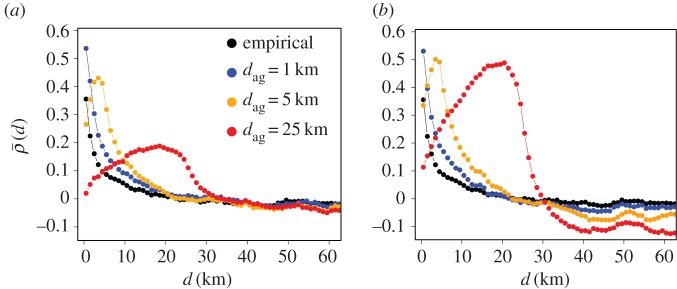
Dependence of the population growth rate in a cell on the population density at distance *d*, $\bar{\rho }(d)$, calculated for the population change between2000 and 2005. We varied the values of *d*_ag_. (*a*) d-aggregate GM. We set *α*=0.8, *β*=0.4 and *γ*=1.0. (*b*) s-aggregate GM. We set *α*=0.4, *β*=1.2 and *γ*=1.0.

**Figure 13.**
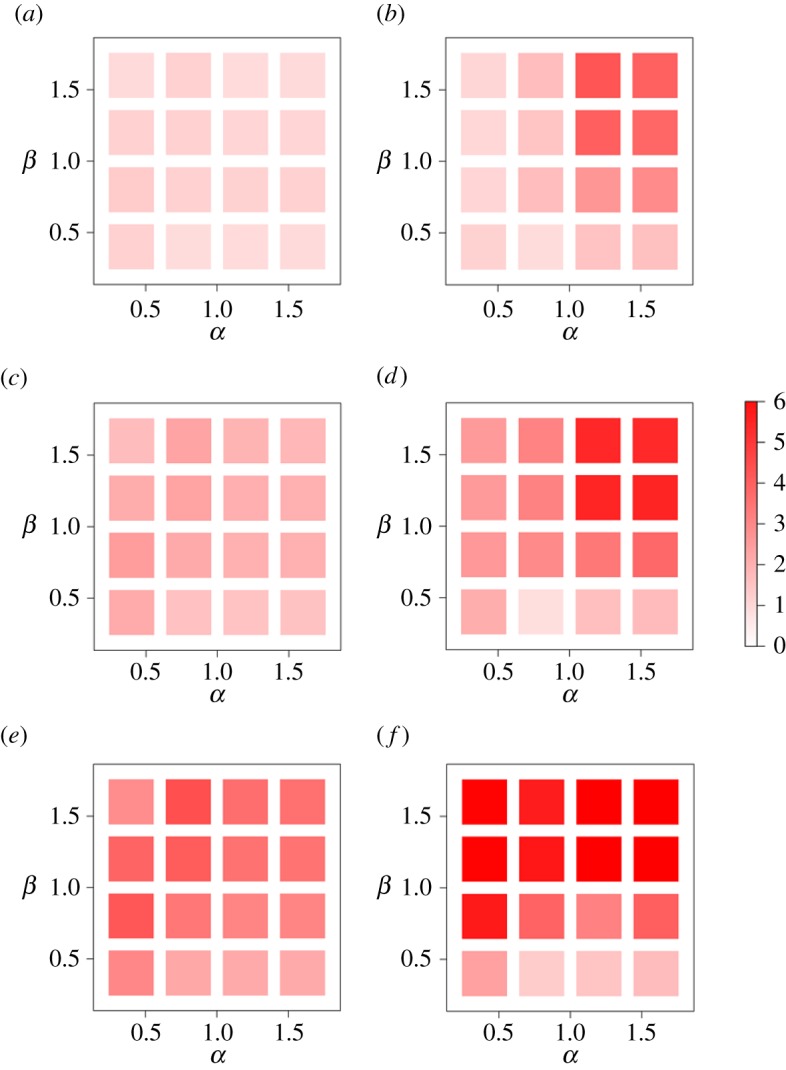
The discrepancy of d-aggregate GM and s-aggregate GM from the empirical data for the population change between 2000 and 2005. (*a*) d-aggregate GM, *d*_ag_=1 km. (*b*) s-aggregate GM, *d*_ag_=1 km. (*c*) d-aggregate GM, *d*_ag_=5 km. (*d*) s-aggregate GM, *d*_ag_=5 km. (*e*) d-aggregate GM, *d*_ag_=25 km. (*f*) s-aggregate GM, *d*_ag_=25 km.

**Figure 14.**
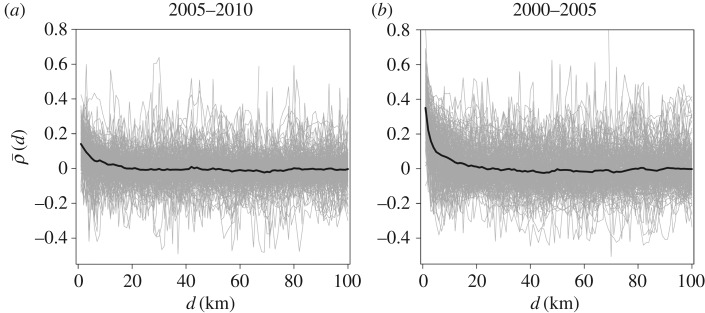
Dependence of the population growth rate in a cell on the population density at distance *d*, *ρ*_*k*_(*d*), calculated from the empirical data. A thin line represents *ρ*_*k*_(*d*) for a region of size 50×50 km. The results for the different regions are superposed on top of each other.The thick lines represent $\bar{\rho }(d)$, which is the average of *ρ*_*k*_(*d*) over all the regions. The thick lines in (*a*) and (*b*) are the same as the lines with the black circles shown in figures 4 and 10, respectively. (*a*) (*t*_1_,*t*_2_)=(2005,2010). (*b*) (*t*_1_,*t*_2_)=(2000,2005).

**Figure 15.**
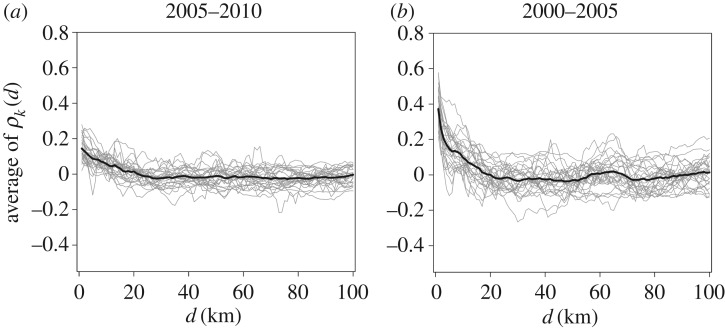
Dependence of the population growth rate in a cell on the population density at distance *d*, *ρ*_*k*_(*d*), calculated from the empirical data. We calculated *ρ*_*k*_(*d*) for regions *k* such that all cells within 30 km from any cell in region *k* do not contain sea. A thin line represents *ρ*_*k*_(*d*) for such a 50×50 km region. The results for the different regions are superposed on top of each other. The thicklines represent the average of *ρ*_*k*_(*d*) over all the regions satisfying the aforementioned criterion. (*a*) (*t*_1_,*t*_2_)=(2005,2010). (*b*) (*t*_1_,*t*_2_)=(2000,2005).

**Figure 16.**
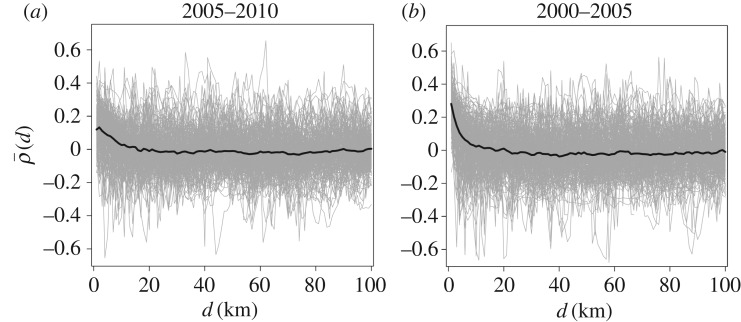
Dependence of the growth rate in a cell on the population density at distance *d*, *ρ*_*k*_(*d*), when only the cells whose number of inhabitants is greater than 100 are considered. A thinline represents *ρ*_*k*_(*d*) for a region of size 50× 50 km. The thick lines represent $\bar{\rho }(d)$, which is the average of *ρ*_*k*_(*d*) over all the regions. (*a*) (*t*_1_,*t*_2_)=(2005,2010). (*b*) (*t*_1_,*t*_2_)=(2000,2005).

**Figure 17.**
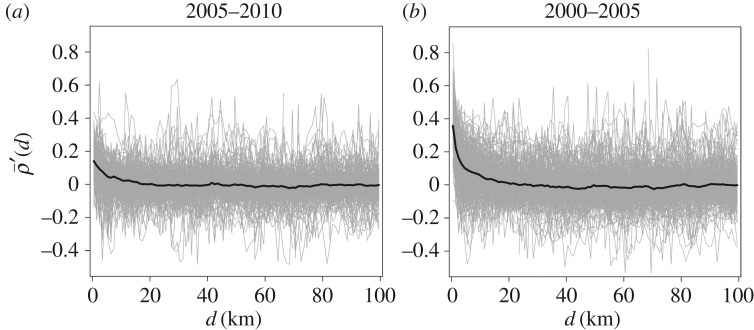
Dependence of the growth rate in a cell on the population density at distance *d*, $\rho_{k}^{\prime }(d)$, controlling for the population size of a focal cell. A thin line represents $\rho_{k}^{\prime }(d)$ for aregion. The thick lines represent ${\bar{\rho }}^{\prime }(d)$, which is the average of $\rho_{k}^{\prime }(d)$ over all the regions. (*a*) (*t*_1_,*t*_2_)=(2005,2010). (*b*) (*t*_1_,*t*_2_)=(2000,2005).

## Data accessibility

Data are available from http://e-stat.go.jp/SG2/eStatGIS/page/download.html.

## Appendix A. Population changes between 2000 and 2005

In the main text, we used the data on the population census in Japan in 2005 and 2010. The data on 2000 are also publicly available although they are less accurate than those in 2005 and 2010 [30]. Here, we set *t*_1_=2000 and *t*_2_=2005 and ran the same analysis pipeline with that in the main text to examine the robustness of our results. As shown in the following, the results were qualitatively the same as those shown in the main text for (*t*_1_,*t*_2_)=(2005,2010) (figures 4–7), except for the behaviour of the GM.

In figure 10, $\bar{\rho }(d)$ obtained from the empirical data, the GM, d-aggregate GM and s-aggregate GM is compared. Similar to the analysis shown in the main text, for the three GMs, we set *γ*=1 and varied *α*, *β*∈{0.4,0.8,1.2,1.6} and used the optimized parameter values. The $\bar{\rho }(0)$ value for the GM was negative, contradicting the empirical data, whereas the behaviour of the d-aggregate and s-aggregate GMs was qualitatively the same as that of the empirical data.

For *α*∈{0.4,0.8,1.2,1.6} and *β*∈{0.4,0.8,1.2,1.6}, the discrepancy between the model and empirical data (equation (2.8)) is shown in figure 11. The results for the GM were inaccurate for all parameter combinations that we considered (figure 11*a*). The d-aggregate GM yielded a good agreement with the data in a wide parameter region (figure 11*b*). The s-aggregate GM was accurate only for *α*=0.4 (figure 11*c*). These results are similar to those for (*t*_1_,*t*_2_)=(2005,2010) (figure 5).

We then examined the robustness of the results with respect to the *d*_ag_ value. The discrepancy between the models and the empirical data is shown in figure 12. For both d-aggregate and s-aggregate GMs, $\bar{\rho }(d)$ behaved similarly to that for the empirical data when *d*_ag_=1 km but not when *d*_ag_=5 km and 25 km. Figure 13 confirms this result for various values of *α* and *β*. For a wide region of the *α*–*β* parameter space, the discrepancy increased as *d*_ag_ increased.

## Appendix B. Plot of *ρ*_*k*_(*d*) for each 50×50 km region

In the main text, we showed the values of *ρ*_*k*_(*d*) averaged over all regions of size 50×50 km, denoted by $\bar{\rho }(d)$ (figure 4). The *ρ*_*k*_(*d*) for each region *k* is plotted as a function of *d* in figure 14. The values of *ρ*_*k*_(*d*) depend considerably on the region.

To calculate $\bar{\rho }(d)$, we used all regions. However, some regions and their nearby regions include water-surface cells, potentially biasing the estimation of $\bar{\rho }(d)$. Therefore, we examined the *ρ*_*k*_(*d*) values for region *k* such that all cells within region *k* and those within 30 km from any cell in region *k* are not in the sea. The average of *ρ*_*k*_(*d*) over these regions is qualitatively the same as that shown in the main text (figure 15).

## Appendix C. Analysis of cells with more than 100 inhabitants

In the main text, we used cells whose population size was between 10 and 100. Figure 16 shows $\bar{\rho }(d)$ for cells whose population size was greater than 100. The behaviour of $\bar{\rho }(d)$ was qualitatively the same as that for the cells of the population size between 10 and 100 (figure 4).

## Appendix D. Analysis with the partial correlation coefficient

In the main text, we calculated *ρ*_*k*_(*d*) using the Pearson correlation coefficient (equation (4)). However, the strong spatial correlation in the population size combined with the tendency that a highly populated cell grows more than sparsely populated cells do may result in spuriously large *ρ*_*k*_(*d*) values. Therefore, to control for the spatial correlation in the population size, we calculated the partial correlation coefficient between the population growth rate of a cell and the population density in nearby cells, $\rho_{k}^{\prime }(d)$, by

D 1 $$\rho_{k}^{\prime }(d)=\frac{\rho_{k}(d)-{\mathrm{cor}}_{k}(D_{i}(d),n_{i})\times {\mathrm{cor}}_{k}(R_{i},n_{i})}{\sqrt{1-{\mathrm{cor}}_{k}(D_{i}(d),n_{i})}\sqrt{1-{\mathrm{cor}}_{k}(R_{i},n_{i})}},$$

where cor_*k*_(⋅,⋅) is the Pearson correlation coefficient between two variables in region *k*; *D*_*i*_(*d*) is the population density averaged over the cells at distance *d* from cell *i* in region *k*; *R*_*i*_ is the population growth rate of cell *i* in region *k*; and *n*_*i*_ is the number of inhabitants in cell *i* in region *k*. We defined ${\bar{\rho }}^{\prime }(d)$ as the average of $\rho_{k}^{\prime }(d)$ over all regions. Figure 17 shows that ${\bar{\rho }}^{\prime }(d)$ as a function of *d* behaves similarly to $\bar{\rho }(d)$ (figure 4).

## Appendix E. Gravity model with the population density aggregated around both the source and destination cells

In the main text, we aggregated the cells around either the source or destination cell but not both. Here, we carried out aggregation around both the source and destination cells. In the model, which we refer to as the GM with the aggregation around both the source and destination (sd-aggregate GM), the population flow from cell *i* to cell *j* is defined by

E 1 $$T_{ij}=Gn_{i}\frac{N_{i}{\left(d_{\mathrm{ag}}\right)}^{\alpha -1}N_{j}{\left(d_{\mathrm{ag}}\right)}^{\beta }}{d_{ij}^{\gamma }}.$$

As we present in the following, the behaviour of the sd-aggregate GM was similar to that of the d-aggregate GM (figures 4–7).

We compare $\bar{\rho }(d)$ between the empirical and simulated data in figure 18. The behaviour of $\bar{\rho }(d)$ obtained from the GM was qualitatively the same as that of the empirical data. The discrepancy between the model and empirical data (equation (2.8)) was small in a wide parameter region (figure 19). We also confirmed that the discrepancy increased as *d*_ag_ increased (figures 20 and 21). The net flow, inflow and outflow for the sd-aggregate GM simulated on a chain with 21 cells are shown in figure 22. The inflow and outflow for the sd-aggregate GM (figure 22*b*,*c*) were similar to those for the d-aggregate GM (figure 9*e*) and the s-aggregate GM (figure 9*i*), respectively. As a result, the net flow for the sd-aggregate GM (figure 22*a*) was similar to that for the d-aggregate GM (figure 9*d*) and the s-aggregate GM (figure 9*g*).
